# Author Correction: A pandemic-enabled comparison of discovery platforms demonstrates a naïve antibody library can match the best immune-sourced antibodies

**DOI:** 10.1038/s41467-022-29876-3

**Published:** 2022-04-12

**Authors:** Fortunato Ferrara, M. Frank Erasmus, Sara D’Angelo, Camila Leal-Lopes, André A. Teixeira, Alok Choudhary, William Honnen, David Calianese, Deli Huang, Linghan Peng, James E. Voss, David Nemazee, Dennis R. Burton, Abraham Pinter, Andrew R. M. Bradbury

**Affiliations:** 1 Specifica Inc, Santa Fe, NM 87505 USA; 2 Bioscience Division, New Mexico Consortium, Los Alamos, NM 87544 USA; 3 Public Health Research Institute, New Jersey Medical School, Rutgers, The State University of New Jersey, Newark, NJ 07103 USA; 4 Life Sciences Institute, Zhejiang University, Hangzhou, China; 5 Department of Immunology and Microbiology, The Scripps Research Institute, La Jolla, CA 92037 USA; 6 Ragon Institute of MGH, MIT and Harvard, Cambridge, MA 02139 USA

**Keywords:** Applied immunology, Antibody generation, SARS-CoV-2, Viral infection

Correction to: *Nature Communications* 10.1038/s41467-021-27799-z, published online 24 January 2022.

The original version of this Article contained errors in References 1, 33 and 77.

Ref. 1 was incorrectly given with incomplete bibliographical information as: ‘Jackson, L. A. et al. An mRNA Vaccine against SARS-CoV-2 – Preliminary Report. *N. Engl. J. Med.* (2020)’. The correct form of Ref. 1 is: ‘Jackson, L. A. et al. An mRNA vaccine against SARS-CoV-2—preliminary report. *N. Engl. J. Med*. **383**, 1920–1931 (2020)’.

Ref. 33 was incorrectly given with incomplete patent information as: ‘Bradbury, A. R. M., Erasmus, M. F. & Teixeira, A. (ed. USPTO) (Specifica Inc, US; 2020)’. The correct form of Ref. 33 is: ‘Bradbury, A. R. M., Erasmus, M. F. & Teixeira, A. A. R. US patent 10,954,508 (2021)’.

Ref. 77, which was given as: ‘77. Kreye, J. et al. A Therapeutic Non-self-reactive SARS-CoV-2 Antibody Protects from Lung Pathology in a COVID-19 Hamster Model. *Cell* **183**, 1058–1069 e1019 (2020).’, was a duplication of Ref. 48. In the correct version, the duplicate reference is removed and all other references are renumbered accordingly.

In addition, the original version of this Article contained an error in the fourth sentence of the Discussion, which incorrectly read ‘Presently, 86 vaccines against 18 different organisms (including strains) are approved for use in the US’ The correct version replaces this sentence with ‘Presently, 103 vaccines against 32 different organisms (including strains) are approved for use in the US’.

This has been corrected in both the PDF and HTML versions of the Article.

